# Classification of Pigeonpea (*Cajanus cajan* (L.) Millsp.) Genotypes for Zinc Efficiency

**DOI:** 10.3390/plants9080952

**Published:** 2020-07-28

**Authors:** Sanjib Kumar Behera, Arvind Kumar Shukla, Pankaj Kumar Tiwari, Ajay Tripathi, Pooja Singh, Vivek Trivedi, Ashok Kumar Patra, Soumitra Das

**Affiliations:** 1 ICAR-Indian Institute of Soil Science, Bhopal, Madhya Pradesh 462 038, India; sanjibkumarbehera123@gmail.com (S.K.B.); pankajkumar2311@gmail.com (P.K.T.); tripathiajay17@gmail.com (A.T.); patraak@gmail.com (A.K.P.); 2 Rajmata Vijayaraje Scindia Krishi Viswavidyalaya, Gwalior, Madhya Pradesh 474 002, India; singh_pooja883@yahoo.co.in; 3 Division of Soil Science and Agricultural Chemistry, ICAR-Indian Agricultural Research Institute, New Delhi 110012, India; vivektrivedi002@gmail.com; 4 International Zinc Association, New Delhi 110062, India; sdas@zinc.org

**Keywords:** pigeonpea, vertisol, soil zinc, seed zinc concentration, biofortification

## Abstract

Pigeonpea (*Cajanus cajan* (L.) Millsp.) is grown globally for its protein-rich seed. However, low availability of soil zinc (Zn) adversely affects the seed yield of pigeonpea. The present study was therefore conducted to assess the Zn efficiency of pigeonpea genotypes based on seed yield and seed Zn uptake efficiency. Field experiments were conducted at the Indian Council of Agricultural Research–Indian Institute of Soil Science, Bhopal, India with twenty different pigeonpea genotypes and two levels of Zn application under a split-plot design. The two levels of Zn were low (without application of Zn fertilizer) and high (with application of 20 kg Zn ha^−1^ (as ZnSO_4_∙7H2O) as basal soil application, in conjunction with three foliar sprays of 0.50% (*w*/*v*) ZnSO_4_∙7H_2_O aqueous solution) (with 0.25% lime as neutralizing agent) at flowering, pod formation, and pod filling stages). Application of Zn improved plant height, branches plant^−1^, pods plant^−1^, seeds pod^−1^, and 100 seed weight of pigeonpea genotypes differently. The mean seed yield, seed Zn concentration, and seed Zn uptake of the genotypes increased from 1.71 to 2.12 t ha^−1^, 32.4 to 43.0 mg kg^−1^, and 54.9 to 90.6 g ha^−1^, respectively, with application of Zn. The seed yield efficiency index (SYEI) and Zn uptake efficiency index (ZUEI) of pigeonpea genotypes varied from 67.0 to 92.5 and from 47.0 to 69.9, respectively. Based on SYEI and ZUEI, the genotypes were classified as efficient and responsive (Virsa Arhar-1, GT-1, GT-101, SKNP 05-05, BDN-2, AAUT 2007-04, BSMR 853, T 15-15, DT 23, Pusa 9), efficient and non-responsive (ICPL 87119, PKV Trombay), inefficient and responsive (AKT 8811, Hisar Paras), and inefficient and non-responsive (AAUT 2007-10, JKM 7, Hisar Manak, C 11, Hisar HO2-60, GAUT 93-17). The efficient and responsive genotypes are the most useful as they yield well under low soil Zn conditions and also respond to Zn fertilizer application. The inefficient and responsive genotypes could be utilized for plant breeding programs by plant breeders for identification and utilization of responsive traits.

## 1. Introduction

Pigeonpea (*Cajanus cajan* (L.) Millsp.), a protein rich legume crop, is cultivated in tropical and subtropical regions of the world. It is a vital grain legume crop in several countries of Asia, Africa, and Latin America. The largest share (≈75%) of global pigeonpea production comes from India. Other major pigeonpea producing countries are Myanmar, Tanzania, Malawi, Kenya, and Uganda. In India, pigeonpea is cultivated in an area of 3.96 million ha [1] located mainly in Karnataka, Maharashtra, Madhya Pradesh, Gujarat, Uttar Pradesh, Odisha, Jharkhand, Andhra Pradesh, and Telangana states. It is the second largest pulse crop in India and the dried split-seeds are consumed by the majority of the Indian population as a source of protein.

Wide-spread Zn deficiency exists in different soils of the world, including those of India [2,3]. Low availability of soil zinc (Zn) adversely affects plant growth parameters such as plant height, number of branches, pod number, seed yield, and Zn concentration of seed and tissue of pigeonpea [4,5] due to reduced enzyme activity influencing the plant metabolism [6]. Zinc malnutrition is also a major micro-nutritional problem in the global population, particularly associated with women and children of developing countries [7]. Enhancing seed yield and seed Zn concentration of food grain meant for human consumption may alleviate Zn malnutrition. Agronomic biofortification practices, such as addition of Zn fertilizers, either to soil and/or foliar, are generally followed to mitigate soil Zn deficiency and enhance plant growth and yield, and grain Zn concentration and uptake [8]. Compared to Zn application to soil, application of Zn to foliar or both soil and foliar were found to be more effective for augmenting Zn uptake by crop seed and/or grain [9,10,11].

Identification and utilization of Zn efficient crop genotypes is needed for both low- and high-input systems of modern agriculture to enhance crop productivity and reduce cost of cultivation and adverse environmental impacts [12]. The responses of crop genotypes to Zn application vary because of variation in zinc efficiency (ZE) of the genotypes, which is due to differences in root architecture, nature and amount of organic acid released, and uptake and translocation of Zn [13]. Several researchers have reported variations in responses to Zn application by the genotypes of pigeonpea [14,15,16], cowpea (*Vigna unguiculata* L.), and bean (*Phaseolus vulgaris* L.) [17,18,19]. Some genotypes yield well under Zn deficient conditions, whereas some genotypes respond well to the external application of Zn fertilizer. The better performance of Zn efficient crop genotypes is because of their ability to tolerate Zn deficiency conditions by utilizing native soil Zn. In contrast, the responsive genotypes absorb and utilize applied Zn efficiently. Thus, there is need for classification of pigeonpea genotypes based on ZE either for obtaining higher seed yield, higher Zn density in seed, or for both higher grain yield and seed Zn density depending on the Zn application strategies.

Zinc efficiency of wheat genotypes, grown under field conditions, was assessed based on grain yield [20], and both grain yield and grain Zn uptake (Singh et al., 2020), for their classification into different groups based on ZE. Similarly, wheat genotypes were also evaluated for Mn efficiency based on grain yield and Mn uptake by grain for their classification into different groups of Mn efficiency [21]. However, the responses of pigeonpea genotypes to Zn application and their classification into different classes of ZE are not well documented. It was, therefore, hypothesized that the genotypes of pigeon pea respond differently to Zn application and the ZE of genotypes is different. The present experiment was, therefore, undertaken to assess the effect of Zn application on growth parameters (plant height, branches plant^−1^, pods plant^−1^, seeds pod^−1^, and 100 seed weight), seed yield, and seed Zn concentration and uptake by pigeonpea genotypes, and to classify the genotypes based on seed yield and seed Zn uptake efficiency. This will help farmers, farm managers, and plant breeders utilize suitable pigeonpea genotypes based on their ZE, as per their requirements.

## 2. Results

The ANOVA for plant height, branches plant^−1^, pods plant^−1^, seeds pod^−1^, 100 seed weight, seed yield, seed Zn concentration, and seed Zn uptake of pigeonpea genotypes indicated a significant effect of Zn treatments and pigeonpea genotypes in both years (Table 1). Therefore, the data for two years were pooled for statistical analysis.

### 2.1. Plant Height, Branches Plant^−1^, Pods Plant^−1^, Seeds Pod^−1^, and 100 Seed Weight of Pigeonpea Genotypes

**Figure 1:**
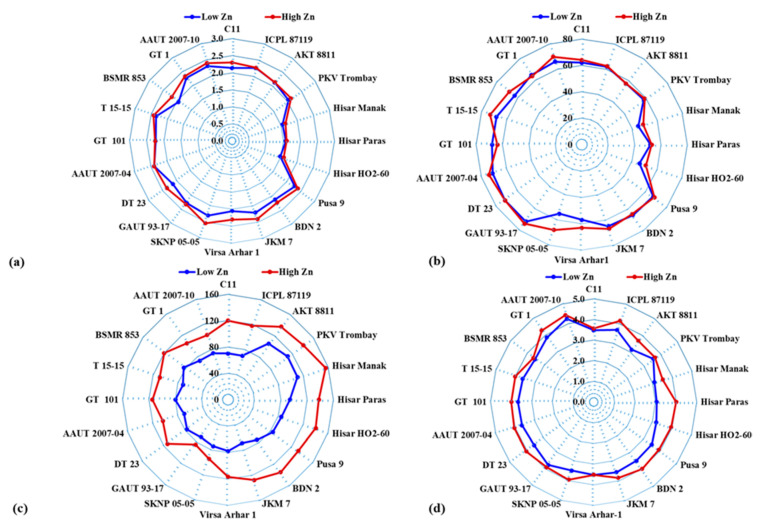
Radar diagrams showing mean (**a**) plant height (m), (**b**) branches plant^−1^, (**c**) pods plant^−1^, and (**d**) seed pod^−1^ of pigeonpea genotypes grown under low and high zinc (Zn) treatments.

Application of Zn influenced plant height, branches plant^−1^, pods plant^−1^, seeds pod^−1^, and 100 seed weight of pigeonpea genotypes (Table 2, Figure 1). Zinc application enhanced mean plant height of pigeonpea genotypes from 2.10 to 2.21 m (Table 2). Under low Zn supply, plant height of the genotypes varied from 1.48 m (Hisar HO2-60) to 2.40 m (AAUT 2007-04). However, the plant height of the genotypes varied from 1.59 m (Hisar HO2-60) to 2.54 m (SKNP 05-05) under high Zn supply (Figure 1). The mean value of the number of branches plant^−1^ increased from 62 to 64 following Zn application. The number of branches plant^−1^ varied from 45 (Hisar Manak) to 72 (GAUT 93-17, DT 23) and from 49 (Hisar Manak) to 74 (GAUT 93-17, AAUT 2007-04) under low and high Zn supply, respectively. The number of pods plant^−1^ of the genotypes varied from 69 (JKM 7) to 112 (PKV Trombay) and from 84 (GAUT 93-17) to 156 (Hisar Manak) under low and high Zn supply, respectively. Application of Zn increased the mean number of pods plant^−1^ from 81 to 121. On average, GAUT 93-17 and Hisar Manak had the lowest (77) and the highest (134) number of pods plant^−1^, respectively. Application of Zn enhanced the mean number of seeds pod^−1^ from 3.53 to 3.90. The number of seeds pod^−1^ of the pigeonpea genotypes varied from 3.05 (Hisar Paras) to 4.24 (AAUT 2007-10) and from 3.52 (Hiasr Mank, Virsa Arhar 1) to 4.43 (AAUT 2007-10) under low and high Zn supply, respectively. On average, Hisar Paras and AAUT 2007-10 had the lowest (3.05) and the highest (4.24) number of seeds pod^−1^, respectively.

### 2.2. Hundred Seed Weight, Seed Yield, Seed Zinc Concentration, and Seed Zinc Uptake of Pigeonpea Genotypes

**Figure 2:**
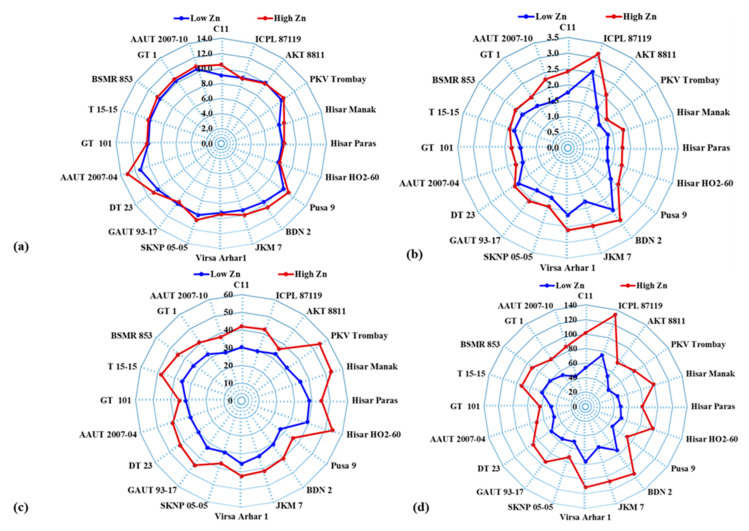
Radar diagrams showing mean (**a**) 100 seed weight (g), (**b**) seed yield (t ha^−1^), (**c**) seed zinc (Zn) concentration (mg kg^−1^), and (**d**) seed Zn uptake (g ha^−1^) of pigeonpea genotypes grown under low and high Zn treatments.

**Figure 3:**
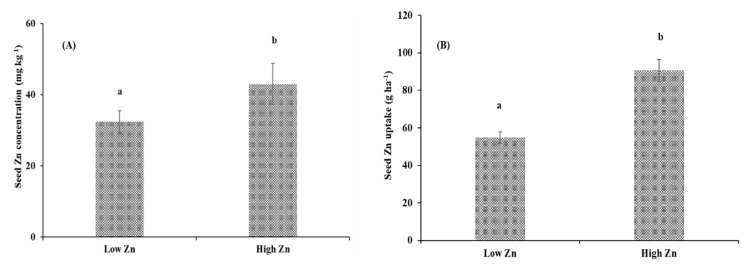
Mean values of (**A**) seed zinc (Zn) concentration (mg kg^−1^) and (**B**) seed Zn uptake (g ha^−1^) of pigeonpea genotypes grown under low and high Zn treatments. Error bars indicate standard deviation of genotypes. Two means having different letters differ significantly.

The weight of 100 seeds of pigeonpea genotypes varied from 8.00 to 11.3 g and from 8.20 to 13.1 g under low and high Zn supply, respectively (Table 3, Figure 2). On average, Hisar HO2-60 and AAUT 2007-04 had the lowest (8.10 g) and the highest (12.2 g) weight of 100 seeds, respectively. The seed yield of the genotypes varied from 1.24 to 2.54 t ha^−1^ and from 1.53 to 3.13 t ha^−1^ under low and high Zn supply, respectively (Table 3). The mean seed yield of the genotypes increased from 1.71 to 2.12 t ha^−1^ with application of Zn. On average, PKV Trombay and ICPL 87119 recorded the lowest (1.39 t ha^−1^) and the highest (2.84 t ha^−1^) seed yield, respectively. Application of Zn enhanced the mean seed Zn concentration of the genotypes from 32.4 to 43.0 mg kg^−1^ (Figure 3). However, seed Zn concentration of the genotypes varied from 27.3 to 39.1 mg kg^−1^ and from 35.2 to 54.6 mg kg^−1^ under low and high Zn supply, respectively. On average, Pusa 9 and Hisar HO2-60 had the lowest (31.6 mg kg^−1^) and the highest (46.6 mg kg^−1^) seed Zn concentration, respectively. The mean value of seed Zn uptake increased from 54.9 to 90.6 g ha^−1^ with Zn application (Figure 3). Seed Zn uptake of the pigeonpea genotypes varied from 39.3 to 75.8 g ha^−1^ and from 62.9 to 133 g ha^−1^ under low and high Zn supply, respectively.

### 2.3. Relationship among Growth and Yield Parameters of Pigeonpea

Pearson’s correlation analysis revealed positive and significant relations of plant height with branches plant^−1^ (r = 0.845, *p* < 0.01), seed pod^−1^ (r = 0.521, *p* < 0.01), 100 seed weight (r = 0.770, *p* < 0.01), and seed yield (r = 0.399, *p* < 0.01) (Table 4). This indicates that the increase in branches plant^−1^, seed pod^−1^,100 seed weight, and seed yield of pigeonpea genotypes occurs with the increase in plant height. There were positive and significant (*p* < 0.01) relations of branches plant^−1^ with seeds pod^−1^ (r = 0.526) and 100 seed weight (r = 0.752). The number of pods plant^−1^ was positively and significantly (*p* < 0.01) correlated with seed Zn concentration (r = 0.753) and seed Zn uptake (r = 0.605). The number of seeds pod^−1^ was significantly and positively correlated with 100 seed weight (r = 0.473, *p* < 0.01), seed yield (r = 0.398, *p* < 0.05), and seed Zn uptake (r = 0.431, *p* < 0.01). This indicates that an increase in seeds pod^−1^ leads to an enhancement in 100 seed weight, seed yield, and seed Zn uptake. There were positive and significant (*p* < 0.01) relations of seed Zn uptake with pods plant^−1^ (r = 0.605), seeds pod^−1^ (r = 0.431), seed yield (r = 0.832), and seed Zn concentration (r = 0.699).

### 2.4. Seed Yield Efficiency Index, Zn Uptake Efficiency Index and Classification of Pigeonpea Genotypes

The SYEI and ZUEI varied significantly among the pigeonpea genotypes (Table 5). Seed yield efficiency index varied from 67.0 ± 1.47 (AAUT 2007-10) to 92.5 ± 1.27 or 1.46 (DT 23, T 15-15) with a mean value of 80.5. The genotypes C 11, AKT 8811, Hisar Manak, Hisar Paras, Hisar HO2-60, JKM 7, and AAUT 2007-10 had lower SYEI values than the mean value of 80.5. The remainder of the genotypes had higher SYEI than the mean value of 80.5. The zinc uptake efficiency index varied from 47.0 ± 2.45 (PKV Trombay) to 69.9 ± 1.18 (SKNP 05-05) with a mean value of 61.2. The genotypes AKT 8811, Hisar Paras, Pusa 9, BDN 2, Virsa Arhar-1, SKNP 05-05, DT 23, AAUT 2007-04, GT 101, T 15-15, BSMR 853, and GT 1 had a higher ZUEI than the mean value.

**Figure 4:**
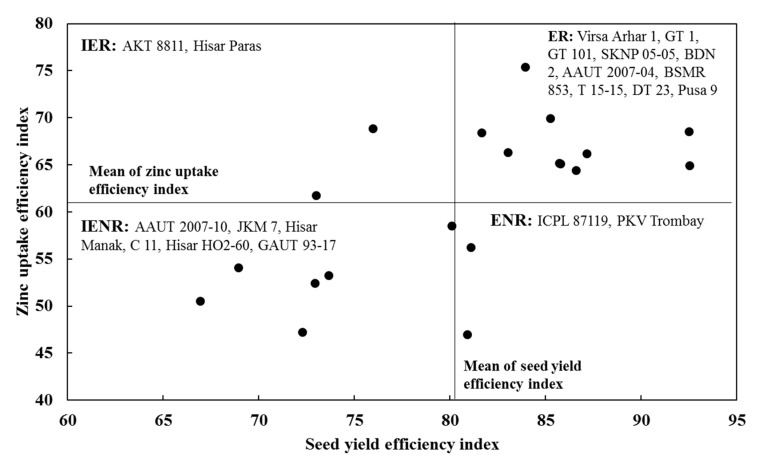
Classification (with 95% confidence interval) of pigeonpea genotypes for zinc (Zn) efficiency. ER = efficient and responsive, ENR = efficient and non-responsive, IER = inefficient and responsive, IENR = inefficient and non-responsive.

The genotypes were classified into four groups based on their SYEI and ZUEI values (Table 6, Figure 4). The mean values of SYEI (80.5) and ZUEI (61.2) were used as criteria to classify the genotypes. The genotypes (Virsha Arhar 1, GT 1, GT 101, SKNP 05-05, BDN 2, AAUT 2007-04, BSMR 853, T 15-15, DT 23, Pusa 9) having higher SYEI and ZUEI were called efficient and responsive (ER). The genotypes AKT 8811 and Hisar Paras, having lower SYEI but higher ZUEI, were called inefficient and responsive (IER). The genotypes (AAUT 2007-10, JKM 7, Hisar Manak, C 11, Hisar HO2-60, and GAUT 93-17) having both lower SYEI and ZUEI were called inefficient and non-responsive (IENR). Genotypes ICPL 87119 and PKV Trombay, having higher SYEI and lower ZUEI, were called efficient and non-responsive (ENR).

## 3. Discussion

Application of Zn increased plant height, branches plant^−1^, pods plant^−1^, seeds pod^−1^, 100 seed weight, seed yield, seed Zn concentration, and seed Zn uptake of pigeonpea genotypes (Table 2 and Table 3). On average, application of Zn enhanced plant height, branches plant^−1^, pods plant^−1^, seeds plant^−1^, and 100 seed weight by 5.24, 1.66, 49.4, 10.5, and 4.66%, respectively. Application of Zn increased mean seed yield, seed Zn concentration, and seed Zn uptake by 24.0, 32.7 and 65.0%, respectively. Other researchers also recorded a positive influence of Zn application on growth and yield parameters of pigeonpea [22,23]. Similar to pigeonpea, green gram crop also responded positively to Zn application [24,25]. Zinc plays an important role in the growth and development of pigeonpea by contributing to the plant metabolism (synthesis of auxin, nucleic acid, and carbohydrate), enzyme activity, stomatal regulation, chlorophyll synthesis, pollen functions, and translocation of photosynthates [26]. Therefore, increase in Zn supply enhanced the growth and yield parameters of pigeonpea. Further, both soil and foliar application of Zn increased seed Zn concentration because of enhanced Zn translocation to seed from leaf and roots [27]. The responses of various pigeonpea genotypes to Zn application were different as shown by variations in enhancement as a percentage of growth and yield parameters. These variations were because of the existence of genetic divergence and phenotypic and genotypic variability among the genotypes [28,29].

The relationship among the growth and yield parameters of pigeonpea (Table 4) reveals that these parameters work in tandem. This finding is in agreement with the results recorded by David et al. [30] who studied 12 vegetable pigeonpea cultivars in eastern Kenya. Udensi et al. [31] also reported positive correlations of plant height with the number of branches and seed yield in soybean. The performance of crop plants depends upon the plant growth and development. Relations among the growth and yield parameters of pigeonpea were also reported by Saxena and Sharma [32], Sodavadiya et al. [33], and Pal et al. [34]. Plant architecture determines the performance of the plant by influencing light interception, photosynthesis, and translocation of photosynthates from source to sink [26]. The information pertaining to the relations of growth parameters with seed yield, seed Zn concentration, and seed Zn uptake could be used in selecting the genotypes for varietal development work.

The seed yield efficiency index and ZUEI were estimated (Table 5) to assess ZE of pigeonpea genotypes. These efficiency indices varied among genotypes. Similarly, ZE and manganese (Mn) efficiency (based on grain yield and Zn and Mn uptake efficiency) of wheat genotypes has also been reported by Singh et al. [35] and Jhanji et al. [21], respectively. The genetic and physiological traits of the genotypes govern ZE, which is also influenced by environmental conditions. The variations in ZE of the pigeonpea genotypes in the present study is corroborated by the differences in growth parameters such as plant height, branches plant^−1^, pods plant^−1^, seeds pod^−1^, and 100 seed weight. Zinc efficient genotypes absorb and utilize Zn more effectively. Therefore, these genotypes could be grown in low soil Zn conditions for increased seed yield and seed Zn uptake to achieve food and nutritional security.

The genotypes DT 23 (92.5) and T 15-15 (92.5) had the highest SYEI and AAUT 2007-10 had the lowest SYEI (AAUT 2007-10). However, GT 101 had the highest (75.4) ZUEI and PKV Trombay had the lowest (47.0) ZUEI. Since both higher seed yield and increased seed Zn uptake are required traits, the pigeonpea genotypes were classified into four groups, namely, ER, IER, IENR, and ENR (Table 6, Figure 4), considering both SEEI and ZUEI. The ER genotypes (Virsa Arhar1, GT-1, GT-101, SKNP 05-05, BDN-2, AAUT 2007-04, BSMR 853, T 15-15, DT 23, and Pusa 9) yield well under low soil Zn conditions but also respond well to application of Zn fertilizer. The IER genotypes (AKT 8811 and Hisar Paras) produce low yield under low soil Zn conditions but respond to Zn fertilizer application. The IENR genotypes (AAUT 2007-10, JKM 7, Hisar Manak, C 11, Hisar HO2-60, and GAUT 93-17) neither yield well under low soil Zn conditions nor respond to Zn fertilizer application. The ENR genotypes (ICPL 87119 and PKV Trombay) yield well under low soil Zn conditions but do not respond to Zn fertilizer application. Both ER and ENR genotypes could be grown by farmers in low soil Zn conditions to obtain better seed yield. In contrast, both ER and IER genotypes could be utilized for agronomic Zn biofortification programs. Since IER genotypes do not yield well under low soil Zn conditions, these genotypes could not be considered by the farmers for cultivation. However, these genotypes could be utilized by plant breeders for plant breeding programs. Moreover, plant breeders could isolate and use the Zn efficiency and Zn responsive traits from the identified genotypes of pigeonpea for developing desired genotypes of pigeonpea and other crops.

## 4. Materials and Methods

### 4.1. Experimental Site

The field experiments were conducted with twenty genotypes of pigeonpea, having different initial seed Zn concentration (Table 7), at the research farm of the Indian Council of Agricultural Research–Indian Institute of Soil Science, Bhopal, India (23° 18′ N latitude, 77° 24′ E longitude and elevation of 485 m) in kharif seasons of 2013–2014 and 2014–2015. The study site experiences a semi-arid and tropical climate. It receives mean annual precipitation of 1005 mm. The major portion of precipitation is received from June to September. The site experiences mean monthly minimum (in the month of January) and maximum (in the month of May) temperatures of 10 and 40 °C, respectively. The soil of the experimental site is hyperthermic *Typic Haplustert* [36]. It is deep black clayey in nature and has a pH of 7.67 [37], electrical conductivity of 0.15 dS m^−1^ [37], organic carbon content of 0.41 (%) [38], available P of 5.20 mg kg^−1^ [39], exchangeable K of 252 mg kg^−1^ [40], exchangeable Ca of 439 mg kg^−1^ [41], exchangeable Mg of 125 mg kg^−1^ [41], CaCl_2_ extractable S of 8.50 mg kg^−1^ [42], diethylenetriaminepenta acetic acid (DTPA) extractable Zn of 0.32 mg kg^−1^ ([43], DTPA extractable Fe of 8.43 mg kg^−1^ [43], DTPA extractable Cu of 1.07 mg kg^−1^ [43], and DTPA extractable Mn of 11.8 mg kg^−1^ [43].

### 4.2. Experimental Details

The experiments were carried out in a split-plot design with Zn application as the main-plot treatment and pigeonpea genotypes as sub-plot treatments. There were two levels (low and high) of Zn treatments and three replications. In low Zn treatment, no Zn fertilizer was applied. Under high Zn treatment, 20 kg Zn ha^−1^ (as ZnSO_4_∙7H_2_O) was applied as basal soil application in combination with three foliar applications of 0.50% (*w*/*v*) ZnSO_4_∙7H_2_O aqueous solution) (with 0.25% lime as neutralizing agent) at flowering, pod formation, and pod filling growth stages [35,44,45,46], The pigeonpea genotypes were cultivated in plots of 6 × 5 m^2^ area, with row to row spacing of 80 cm and plant to plant spacing of 20 cm. The plot of each genotype was separated from the plot of another genotype by 1 m. The crops were supplied with 40 kg of N (as urea) ha^−1^, 80 kg of P_2_O_5_ (as diammonium phosphate) ha^−1^, 30 kg of K_2_O (as muriate of potash) ha^−1^, and 40 kg of S (as bentonite S) ha^−1^ [47]. Hand weeding was carried out as and when required to keep the field weed free.

### 4.3. Recording Observations for Growth Parameters, Plant Sampling and Analysis

At physiological maturity, observations pertaining to growth parameters, such as plant height, branches (primary + secondary) plant^−1^, and pods plant^−1^ were recorded, by averaging the values for 10 plants in each plot. Pods were harvested at physiological maturity and subsequently threshed after sun drying. The number of seeds pod^−1^, 100 seed weight, and seed yield were recorded. For estimating seed Zn concentration, 10 pigeonpea plants were collected from each plot and seeds were separated by hand threshing. Seed samples were washed with double-distilled water three times and then oven dried at 75 °C for 48 h. Dried seed samples were ground in a stainless-steel mill (Micro-mill grinder, model −0210 × 60, Bel-Art- SP Scienceware) to <1 mm. Powdered seed samples were digested in a di-acid mixture (nitric acid and perchloric acid mixture in 9:4 ratio) [48]. The concentration of Zn in digested material was determined by atomic absorption spectrophotometer (Make and model: Varian AA 240 FS). The Zn uptake in pigeonpea seed, seed yield efficiency index (SYEI), and Zn uptake efficiency index (ZUEI) [49] were estimated as described below.

Zn uptake in pigeonpea seed (g ha^−1^) = Seed yield (t ha^−1^) × Seed Zn concentration (mg kg^−1^) (1)

(2) $$\mathrm{Seed}\;\mathrm{yield}\;\mathrm{efficiency}\;\mathrm{index}\;(\mathrm{SYEI})=\frac{\mathrm{Seed}\;\mathrm{yield}\;\mathrm{under}\;\mathrm{low}\;\mathrm{Zn}}{\mathrm{Seed}\;\mathrm{yield}\;\mathrm{under}\;\mathrm{high}\;\mathrm{Zn}}\times 100$$

(3) $$\mathrm{Zn}\;\mathrm{uptake}\;\mathrm{efficiency}\;\mathrm{index}\;(\mathrm{ZUEI})=\frac{\mathrm{Seed}\;\mathrm{Zn}\;\mathrm{uptake}\;\mathrm{under}\;\mathrm{low}\;\mathrm{Zn}}{\mathrm{Seed}\;\mathrm{Zn}\;\mathrm{uptake}\;\mathrm{under}\;\mathrm{high}\;\mathrm{Zn}}\times 100$$

The pigeonpea genotypes were classified into four groups based on their SYEI and ZUEI values. The mean values of SYEI and ZUEI were used as criteria to classify the genotypes [21,35]. Group 1 consisted of efficient and responsive (ER) genotypes having higher efficiency indices than the mean values of SYEI and ZUEI. Group 2 consisted of efficient and non-responsive (ENR) genotypes having higher SYEI but lower ZUEI compared to their respective mean values. Group 3 consisted of inefficient and responsive (IER) with lower SYEI but higher ZUEI. Group 4 had inefficient and non-responsive (IENR) genotypes having lower SYEI and ZUEI than the mean values.

### 4.4. Statistical Analysis

The data were analyzed using analysis of variance (ANOVA) [50] to evaluate the differences between treatment means. The standard errors of differences (SED) were derived from the ANOVA table using the SAS 9.2 software package [51]. Because the least significant difference (LSD) has more power compared to other post hoc comparison methods [52,53], it was used for comparisons where F-probabilities were significant (*p* < 0.05). The year-wise analysis of data was carried out and then pooled for two years as the year-wise variations in data were found to be non-significant.

## 5. Conclusions

Increased Zn supply improved growth parameters, seed yield, seed Zn concentration, and seed Zn uptake of pigeonpea genotypes. Zinc efficiency measured in terms of seed yield efficiency and seed Zn uptake efficiency of genotypes differed significantly. The genotypes could be classified into four groups, namely, efficient and responsive, inefficient and responsive, inefficient and non-responsive, and efficient and non-responsive. Both efficient and responsive and efficient and non-responsive genotypes can be suggested for farmers for cultivation to obtain higher seed yield under low soil Zn conditions. The inefficient and responsive genotypes could be used for plant breeding programs for isolation of genes accountable for the response to Zn fertilizer application. The inefficient and non-responsive genotypes could be improved by plant breeding programs by incorporating efficiency and responsive traits. The genotypes of other important crops could be assessed for Zn efficiency for their classification into different groups and use by various stakeholders.

## Figures and Tables

**Table 1:** Analyses of variance for plant height (m), branches plant^−1^, pods plant^−1^, seeds pod^−1^, 100 seed weight (g), seed yield (t ha^−1^), seed zinc (Zn) concentration (mg kg^−1^), and seed Zn uptake (g ha^−1^) of pigeonpea genotypes for two years separately and pooled. F-ratio indicates ratio of two variances, LSD = least significant difference, CV = coefficient of variation, NS = not significant at *p* < 0.05, * = significant at *p* < 0.05.

Parameters	Factors	Plant Height			Branches Plant^−1^			Pods Plant^−1^		
		First Year	Second Year	Pooled	First Year	Second Year	Pooled	First Year	Second Year	Pooled
F-ratio (calculated)	Year	-	-	0.43	-	-	0.75	-	-	0.62
	Zinc level (Zn)	0.25	0.23	0.50	135.1	146.2	254.3	114.2	132.5	217.8
	Genotypes (G)	0.26	0.25	0.49	0.35	8.24	9.36	1.21	5.86	6.25
	Zn x G	0.32	0.41	0.11	0.43	1.58	0.67	0.25	1.11	0.87
LSD (*p* < 0.05)	Year	-	-	NS	-	-	NS	-	-	NS
	Zinc level (Zn)	*	*	*	*	*	*	*	*	*
	Genotypes (G)	*	*	*	*	*	*	*	*	*
	Zn x G	NS	NS	NS	NS	NS	NS	NS	NS	NS
CV%	Year	-	-	5.84	-	-	7.75	-	-	9.35
	Zinc level (Zn)	10.1	14.2	11.5	8.94	8.51	9.58	8.54	13.21	12.14
	Genotypes(G)	9.89	13.2	12.5	10.5	11.5	12.7	15.2	13.8	14.8
		**Seeds pod^−1^**			**100 seed weight**			**Seed yield**		
		**First year**	**Second year**	**Pooled**	**First year**	**Second year**	**Pooled**	**First year**	**Second year**	**Pooled**
F-ratio (calculated)	Year	-	-	0.42	-	-	0.35	-	-	0.15
	Zinc level (Zn)	0.21	0.19	0.35	0.31	0.25	0.34	0.12	0.21	0.22
	Genotypes (G)	0.31	0.24	0.37	0.38	0.30	0.40	0.15	0.20	0.27
	Zn x G	0.41	0.35	0.17	0.45	0.47	0.25	0.28	0.27	0.31
LSD (*p* < 0.05)	Year	-	-	NS	-	-	NS	-	-	NS
	Zinc level (Zn)	*	*	*	*	*	*	*	*	*
	Genotypes (G)	*	*	*	*	*	*	*	*	*
	Zn x G	NS	NS	NS	NS	NS	NS	NS	NS	NS
CV%	Year	-	-	9.85	-	-	10.1	-	-	12.4
	Zinc level (Zn)	10.1	10.5	9.65	9.85	10.1	10.5	11.4	10.8	14.9
	Genotypes(G)	8.59	11.54	10.4	10.4	11.2	12.5	12.4	11.7	13.5
		**Seed Zn concentration**			**Seed Zn uptake**					
		**First year**	**Second year**	**Pooled**	**First year**	**Second year**	**Pooled**			
F-ratio (calculated)	Year	-	-	1.21	-	-	2.56			
	Zinc level (Zn)	71.5	85.4	135.6	114.2	175.3	341.7			
	Genotypes (G)	2.54	3.12	7.45	2.11	5.87	9.14			
	Zn x G	0.34	0.47	0.18	0.58	0.98	0.21			
LSD (*p* < 0.05)	Year	-	-	NS	-	-	NS			
	Zinc level (Zn)	*	*	*	*	*	*			
	Genotypes (G)	*	*	*	*	*	*			
	Zn x G	NS	NS	NS	NS	NS	NS			
CV%	Year	-	-	9.78	-	-	8.74			
	Zinc level (Zn)	7.45	9.13	9.41	10.1	11.5	12.4			
	Genotypes(G)	9.14	10.1	10.8	9.56	10.7	11.9			

**Table 2:** Plant height (m), number of branches per plant, number of pods per plant and number of seeds per plant of pigeon pea genotypes under low and high zinc (Zn) treatments. SED = standard error of difference, LSD = least significant difference. * denotes significant at *p* < 0.05.

Genotypes	Plant Height (m)			Branches Plant^−1^			Pods Plant^−1^			Seeds Pod^−1^		
	Low Zn	High Zn	Mean	Low Zn	High Zn	Mean	Low Zn	High Zn	Mean	Low Zn	High Zn	Mean
C11	2.14	2.30	2.22	62	64	63	70	120	95	3.48	3.57	3.48
ICPL 87119	2.25	2.26	2.26	62	62	62	70	118	94	3.67	4.14	3.67
AKT 8811	2.12	2.13	2.13	57	57	57	105	137	121	3.14	3.67	3.14
PKV Trombay	2.04	2.13	2.09	58	59	59	112	141	127	3.57	3.67	3.57
Hisar Manak	1.55	1.64	1.60	45	49	47	111	156	134	3.10	3.52	3.10
Hisar Paras	1.57	1.60	1.59	52	53	53	94	138	116	3.05	4.00	3.05
Hisar HO2-60	1.48	1.59	1.54	46	51	49	85	140	113	3.19	3.95	3.19
Pusa 9	2.27	2.38	2.33	67	68	68	84	132	108	3.48	3.91	3.48
BDN 2	2.13	2.24	2.19	66	65	66	75	136	106	3.52	4.00	3.52
JKM 7	2.21	2.41	2.31	65	67	66	69	128	99	3.57	3.85	3.57
Virsa Arhar1	2.06	2.31	2.19	57	63	60	78	117	98	3.52	3.52	3.52
SKNP 05-05	2.30	2.54	2.42	55	68	62	74	94	84	3.48	3.95	3.48
GAUT 93-17	2.26	2.30	2.28	72	74	73	70	84	77	3.76	3.90	3.76
DT 23	2.15	2.36	2.26	72	72	72	77	114	96	3.57	4.05	3.57
AAUT 2007-04	2.40	2.40	2.40	71	74	73	70	104	87	3.66	4.05	3.66
GT 101	2.24	2.26	2.25	68	64	66	80	115	98	3.67	4.00	3.67
T 15-15	2.34	2.42	2.38	68	73	71	72	109	91	3.62	4.00	3.62
BSMR 853	1.94	2.19	2.07	63	68	66	83	120	102	3.52	3.62	3.52
GT 1	2.28	2.35	2.32	65	64	65	73	106	90	3.86	4.29	3.86
AAUT 2007-10	2.31	2.40	2.36	66	70	68	74	103	89	4.24	4.43	4.24
Mean	2.10	2.21		62	64		81	121		3.53	3.90	
SED												
Zinc level (Zn)	0.03			0.50			0.80			0.08		
Genotype (G)	0.05			0.98			1.05			0.95		
LSD (*p* < 0.05)												
Zinc level (Zn)	*			*			*			*		
Genotype (G)	*			*			*			*		
Zn x G	*			*			*			*		

**Table 3:** Hundred seed weight (g), seed yield (t ha^−1^), seed zinc (Zn) concentration (mg kg^−1^), and seed Zn uptake (g ha^−1^) of pigeon pea genotypes under low and high Zn treatments. SED = standard error of difference, LSD = least significant difference. NS = not significant at *p* < 0.05, * denotes significant at *p* < 0.05.

Genotypes	100 Seed Weight (g)			Seed Yield (t ha^−1^)			Seed Zn Concentration (mg kg^−1^)			Seed Zn Uptake (g ha^−1^)		
	Low Zn	High Zn	Mean	Low Zn	High Zn	Mean	Low Zn	High Zn	Mean	Low Zn	High Zn	Mean
C11	9.10	10.50	9.55	1.76	2.42	2.09	30.2	42.0	36.1	53.2	102	77.4
ICPL 87119	9.20	9.10	9.15	2.54	3.13	2.84	29.4	42.4	35.9	74.6	133	104
AKT 8811	10.0	9.90	9.95	1.58	2.08	1.83	32.7	36.1	34.4	51.6	75.0	63.3
PKV Trombay	9.90	10.3	10.1	1.24	1.53	1.39	31.7	54.6	43.2	39.3	83.6	61.4
Hisar Manak	8.10	8.80	8.45	1.34	1.85	1.59	34.9	53.4	44.2	46.6	98.7	72.7
Hisar Paras	8.20	8.40	8.30	1.26	1.73	1.49	38.3	45.3	41.8	48.3	78.2	63.2
Hisar HO2-60	8.00	8.20	8.10	1.33	1.80	1.56	39.1	54.1	46.6	51.9	97.4	74.7
Pusa 9	10.3	11.1	10.7	1.69	1.98	1.83	27.3	35.9	31.6	46.2	70.9	58.6
BDN 2	9.60	10.5	10.0	2.43	2.83	2.63	30.5	40.2	35.4	74.1	114	94.0
JKM 7	9.30	10.0	9.65	1.79	2.60	2.20	32.7	41.7	37.2	58.6	108	83.5
Virsa Arhar 1	9.20	9.40	9.30	2.13	2.61	2.37	35.6	42.5	39.1	75.8	111	93.3
SKNP 05-05	10.0	10.7	10.3	1.67	1.96	1.81	30.5	37.2	33.9	50.8	72.7	61.8
GAUT 93-17	9.90	9.60	9.75	1.67	2.09	1.88	32.8	44.9	38.9	54.9	93.8	74.4
DT 23	10.4	11.1	10.8	1.93	2.08	2.00	30.1	42.9	36.5	57.9	89.2	73.6
AAUT 2007-04	11.3	13.1	12.2	1.50	1.73	1.61	30.4	40.9	35.7	45.4	70.6	58.0
GT 101	9.70	9.90	9.80	1.50	1.79	1.64	31.6	35.2	33.4	47.4	62.9	55.2
T 15-15	10.0	10.2	10.1	1.79	1.94	1.86	35.4	47.8	41.6	63.4	92.5	77.9
BSMR 853	10.1	10.5	10.3	1.79	2.05	1.92	33.7	44.4	39.1	60.3	91.1	75.7
GT 1	10.3	10.6	10.4	1.64	1.98	1.81	32.5	40.7	36.6	53.4	80.5	66.9
AAUT 2007-10	10.4	10.8	10.6	1.54	2.29	1.92	28.6	37.9	33.3	43.9	86.9	65.4
Mean	9.65	10.1		1.71	2.12		32.4	43.0		54.9	90.6	
SED												
Zinc level (Zn)	0.10			0.11			0.38			0.25		
Genotype (G)	0.15			0.18			1.15			1.05		
LSD (*p* < 0.05)												
Zinc level (Zn)	*			*			*			*		
Genotype (G)	*			*			*			*		
Zn x G	NS			NS			NS			NS		

**Table 4:** Pearson’s correlation coefficients showing relations among plant height, branches plant^−1^, pods plant^−1^, seeds plant^−1^, 100 seed weight, seed yield, seed zinc (Zn) concentration, and seed Zn uptake of pigeonpea genotypes (n = 38). *, ** denote significant at *p* < 0.05 and *p* < 0.01, respectively.

	Plant Height	Branches Plant^−1^	Pods Plant^−1^	Seeds Pod^−1^	100 Seed Weight	Seed Yield	Seed Zn Concentration	Seed Zn Uptake
Plant height	1.000							
Branches plant^−1^	0.845 **	1.000						
Pods plant^−1^	−0.244	-0.310	1.000					
Seeds pod^−1^	0.521 **	0.526 **	0.206	1.000				
100 seed weight	0.770 **	0.752 **	−0.050	0.473 **	1.000			
Seed yield	0.399 *	0.294	0.243	0.398 *	0.113	1.000		
Seed Zn concentration	−0.254	−0.191	0.753 **	0.242	−0.133	0.196	1.000	
Seed Zn uptake	0.158	0.113	0.605 **	0.431 **	0.012	0.832 **	0.699 **	1.000

**Table 5:** Seed yield efficiency index (SYEI) and zinc (Zn) uptake efficiency index (ZUEI) of pigeonpea genotypes. SED = standard error of difference; LSD = least significant difference.

Genotypes	Seed Yield Efficiency Index (Mean ± Standard Error of Mean)	Zn Uptake Efficiency Index (Mean ± Standard Error of Mean)
C11	72.9 ± 1.35	52.4 ± 1.11
ICPL 87119	81.1 ± 0.97	56.2 ± 1.24
AKT 8811	76.0 ± 1.05	68.8 ± 0.86
PKV Trombay	80.9 ± 2.14	47.0 ± 2.45
Hisar Manak	72.3 ± 1.52	47.2 ± 1.58
Hisar Paras	73.0 ± 1.42	61.7 ± 1.32
Hisar HO2-60	73.7 ± 0.89	53.3 ± 1.11
Pusa 9	85.7 ± 1.45	65.2 ± 0.68
BDN 2	85.8 ± 1.63	65.1 ± 1.14
JKM 7	69.0 ± 1.24	54.1 ± 1.63
Virsa Arhar-1	81.7 ± 1.54	68.4 ± 1.25
SKNP 05-05	85.3 ± 1.23	69.9 ± 1.18
GAUT 93-17	80.1 ± 0.86	58.5 ± 1.17
DT 23	92.5 ± 1.27	64.9 ± 2.14
AAUT 2007-04	86.6 ± 1.12	64.4 ± 1.14
GT 101	83.9 ± 2.14	75.4 ± 1.58
T 15-15	92.5 ± 1.46	68.5 ± 1.43
BSMR 853	87.2 ± 0.96	66.2 ± 1.34
GT 1	83.1 ± 1.27	66.3 ± 1.15
AAUT 2007-10	67.0 ± 1.47	50.5 ± 1.14
Mean	80.5	61.2
SED	1.6	1.8
LSD (*p* < 0.05)	4.53	3.73

**Table 6:** Categories of pigeonpea genotypes for zinc (Zn) efficiency.

Efficient and Responsive (ER)	Efficient and Non-Responsive (ENR)	Inefficient and Responsive (IER)	Inefficient and Non-Responsive (IENR)
Virsa Arhar1, GT-1, GT-101, SKNP 05-05, BDN-2, AAUT 2007-04, BSMR 853, T 15-15, DT 23, Pusa 9	ICPL 87119, PKV Trombay	AKT 8811, Hisar Paras	AAUT 2007-10, JKM 7, Hisar Manak, C 11, Hisar HO2-60, GAUT 93-17

**Table 7:** Initial seed zinc concentration of pigeonpea genotypes used in the study. SED = standard error of difference; LSD = least significant difference.

Genotypes	Grain Zinc Concentration (mg kg^−1^) (Mean ± Standard Error of Mean)
C11	32.0 ± 0.89
ICPL 87119	30.4 ± 0.56
AKT 8811	33.7 ± 1.11
PKV Trombay	33.0 ± 0.98
Hisar Manak	35.5 ± 1.01
Hisar Paras	38.7 ± 0.54
Hisar HO2-60	40.0 ± 1.47
Pusa 9	27.8 ± 0.58
BDN 2	32.0 ± 1.14
JKM 7	33.4 ± 0.97
Virsa Arhar1	37.5 ± 1.52
SKNP 05-05	31.8 ± 1.21
GAUT 93-17	33.1 ± 2.21
DT 23	31.4 ± 1.46
AAUT 2007-04	30.9 ± 0.98
GT 101	32.5 ± 1.18
T 15-15	36.4 ± 1.64
BSMR 853	34.2 ± 1.47
GT 1	33.4 ± 2.10
AAUT 2007-10	29.5 ± 1.15
SED	0.571
LSD	2.065
